# Supplementary material for: Cocaine-induced loss of LTD and social impairments are restored by fatty acid amide hydrolase inhibition
Source: Sci Rep. 2023 Oct 25;13:18229. doi: 10.1038/s41598-023-45476-7 (PMC10600200; doi:10.1038/s41598-023-45476-7)
Supplement: Supplementary file 1 — Supplementary Legends. [file 41598_2023_45476_MOESM1_ESM.docx]

**Cocaine-induced loss of LTD and social impairments are restored by fatty acid amide hydrolase inhibition**

Laia Alegre-Zurano^1^, Alba Caceres-Rodriguez^3,4^, Paula Berbegal-Sáez^1^, Olivier Lassalle^3,4^, Olivier Manzoni^3,4#^ and Olga Valverde^1,2#*^

**SUPPLEMENTARY MATERIAL**

**Supplementary Figure 1. Liquid consumption in the saccharin preference test following cocaine exposure. A** Saccharine, **B** water and **C** total liquid consumption 1 hour following an acute cocaine or saline administration. **D** Saccharine, **E** water and **F** total liquid consumption 24 hours following an acute cocaine or saline administration. Student’s *t* test.

**Supplementary Figure 2. Liquid consumption in the saccharin preference test following cocaine exposure and URB597 treatment.** 24 hours after cocaine or saline exposure, **A** Saccharine, **B** water and **C** total liquid consumption 1 hour following URB597 or vehicle treatment. 24 hours after cocaine or saline exposure, **D** Saccharine, **E** water and **F** total liquid consumption 24 hours following URB597 or vehicle treatment. Two-way ANOVA.
