# Supplementary figures and images for: Cocaine-induced loss of LTD and social impairments are restored by fatty acid amide hydrolase inhibition
Source: Sci Rep. 2023 Oct 25;13:18229. doi: 10.1038/s41598-023-45476-7 (PMC10600200; doi:10.1038/s41598-023-45476-7)

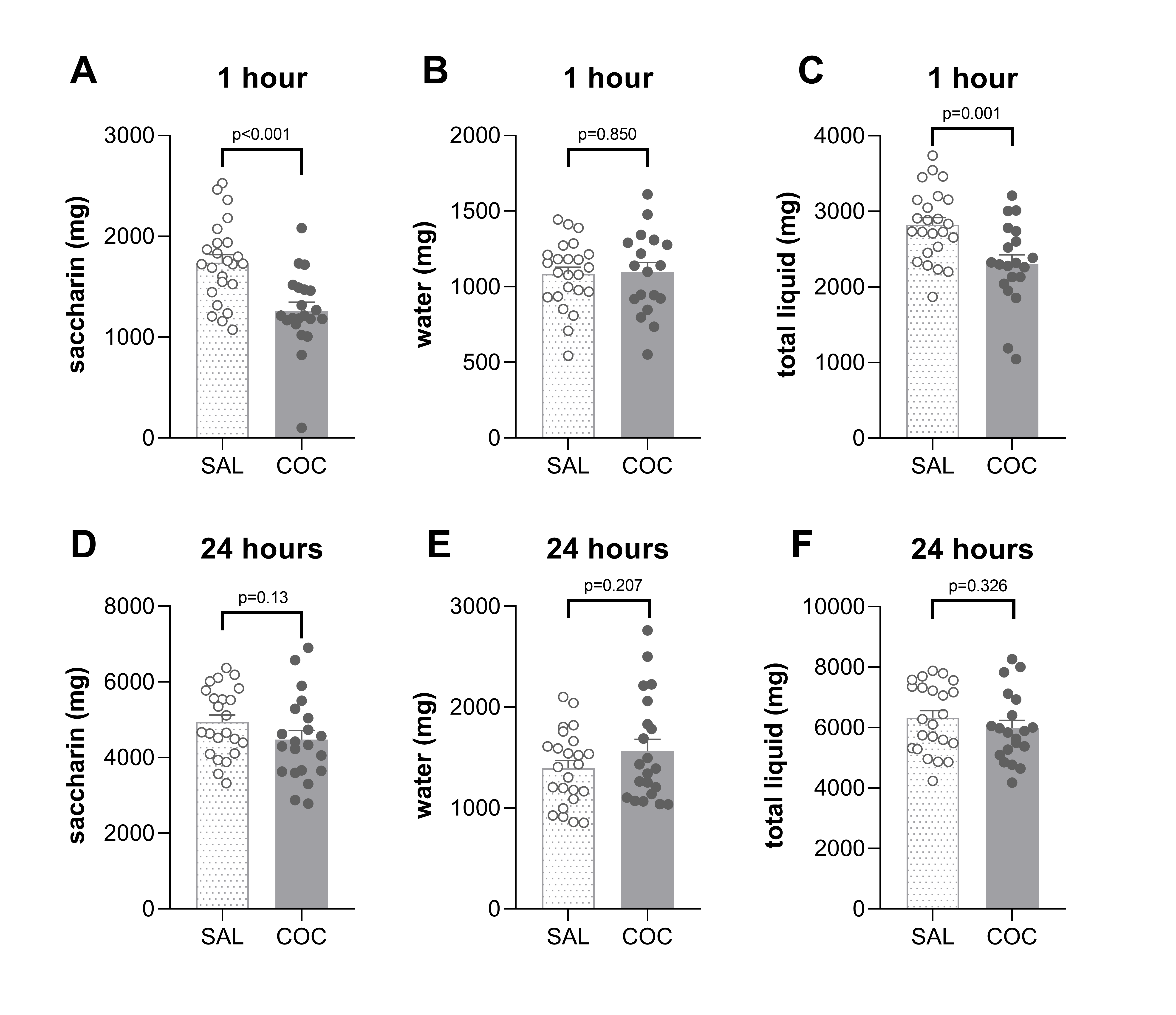

Supplement: Supplementary file 2 — Supplementary Figure 1. [file 41598_2023_45476_MOESM2_ESM.tif]

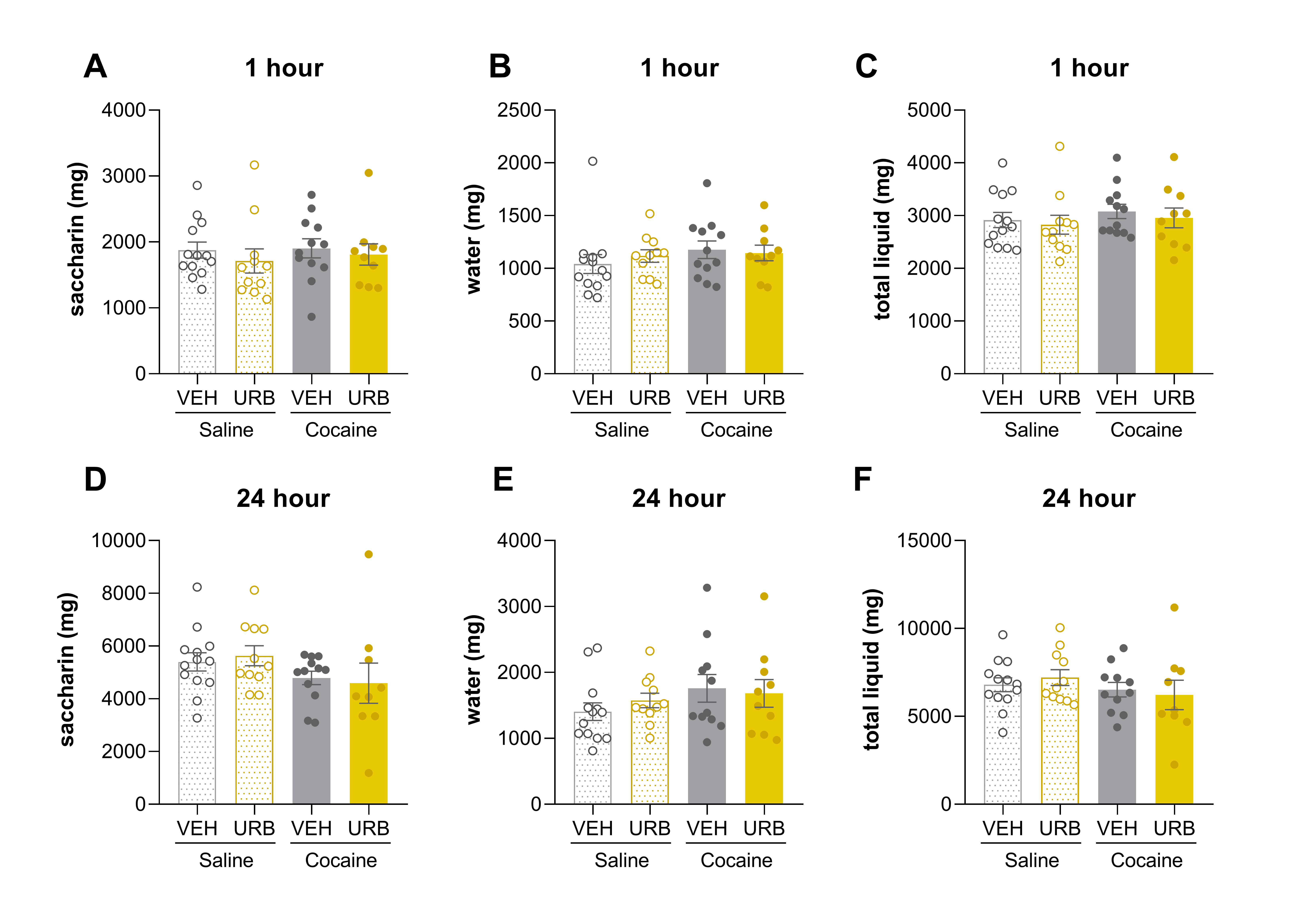

Supplement: Supplementary file 3 — Supplementary Figure 2. [file 41598_2023_45476_MOESM3_ESM.tif]
